# Urinary Sodium Excretion and Adherence to the Mediterranean Diet in Older Adults

**DOI:** 10.3390/nu14010061

**Published:** 2021-12-24

**Authors:** Sara Moreira, Pedro Moreira, Ana S. Sousa, Rita S. Guerra, Cláudia Afonso, Alejandro Santos, Nuno Borges, Teresa F. Amaral, Patrícia Padrão

**Affiliations:** 1 FCNAUP, Faculdade de Ciências da Nutrição e Alimentação, Universidade do Porto, Rua do Campo Alegre, 823, 4150-180 Porto, Portugal; ssfm92@gmail.com (S.M.); sofia.limas.sousa@gmail.com (A.S.S.); ritacsguerra@gmail.com (R.S.G.); claudiafonso@fcna.up.pt (C.A.); alejandrosantos@fcna.up.pt (A.S.); nunoborges@fcna.up.pt (N.B.); tamaral@fcna.up.pt (T.F.A.); patriciapadrao@fcna.up.pt (P.P.); 2 EPIUnit, Instituto de Saúde Pública, Universidade do Porto, Rua das Taipas, 135, 4050-600 Porto, Portugal; 3 Laboratory for Integrative and Translational Research in Population Health (ITR), Rua das Taipas, n.° 135, 4050-600 Porto, Portugal; 4 Centro de Investigação em Atividade Física, Saúde e Lazer, Universidade do Porto, Rua Dr. Plácido da Costa, 4200-450 Porto, Portugal; 5 I3S, Instituto de Investigação e Inovação em Saúde, Rua Alfredo Allen, 4200-135 Porto, Portugal; 6 CINTESIS, Centre for Health Technology and Services Research, Rua Dr. Plácido da Costa, 4200-450 Porto, Portugal; 7 LAETA-INEGI, Faculdade de Engenharia, Universidade do Porto, Rua Dr. Roberto Frias, 4200-465 Porto, Portugal

**Keywords:** elderly, Mediterranean Diet, salt, sodium

## Abstract

Despite the well-known benefits of the Mediterranean Diet (MedDiet), data on the sodium intake is scarce. This study aimed to quantify the association between sodium excretion and the adherence to the MedDiet in the elderly. A representative sample of 1500 Portuguese adults (≥65 years) was assessed (1321 were eligible for the present analysis). A 24 h urine sample was collected and analysed for creatinine and sodium. Excessive sodium intake was defined as above 2000 mg/day. The adherence to the MedDiet was assessed by the PREDIMED. A binary logistic regression model was conducted to evaluate the association between urinary sodium excretion and the adherence to the MedDiet. Odds Ratios (OR) and respective 95% Confidence Intervals (95% CI) were calculated. Excessive sodium excretion was observed in 80.0% of men and 91.5% of women whereas a high adherence to the MedDiet was reported by 42.2% of women and 46.4% of men. After adjusting for confounders, excessive sodium excretion was associated with a high adherence to the MedDiet in men (OR = 1.94; 95% CI: 1.03–3.65) but not in women. These results show that the MedDiet can be an important source of sodium and highlight the need for implementing strategies to reduce sodium intake when following a MedDiet.

## 1. Introduction

The Mediterranean Diet (MedDiet) is characterized by the predominance of foods of plant origin such as vegetables, fruit, bread, potatoes, beans, nuts, and seeds; olive oil as the main source of fat; dairy products (mainly cheese and yogurt); and fish, poultry, and eggs, consumed in low to moderate amounts instead of red meat, and wine consumed in low to moderate amounts, usually with meals [1]. However, the role of salt in this dietary pattern remains unclear.

Although the MedDiet was previously recommended as preventive for the development of several conditions including hypertension [2], it may be a source of hidden sodium both through added salt during cooking or at the table, and also by including non-negligible quantities of processed products that are rich in salt [3]. 

Excessive sodium intake has been associated with hypertension [3,4,5,6,7,8], which is a major risk factor for cardiovascular diseases, contributing to almost half of related deaths [5,8,9]. It was demonstrated that in hypertensive patients, the reduction in salt intake for just two weeks is enough to reduce the global cardiovascular risk [10].

The World Health Organization (WHO) recommends the consumption of no more than two grams of sodium per day (equivalent to five grams of salt (sodium chloride)/day) in adults in order to reduce the burden of non-communicable diseases [11]. However, consumption levels much higher than the recommendations have been reported in several countries around the world [12], including Portugal, where a mean intake of 10.7 g/day of salt was reported in 2012 [13]. In the present study which included a representative sample of Portuguese older adults, the median daily sodium excretion was slightly lower, at 3368 mg, which corresponds to 8.4 g of salt [14]. This issue is of particular importance in Portugal since the overall prevalence of hypertension in adults was 36.0% in 2015, although the estimate is much higher among individuals older than 64 years, for whom a prevalence of 71.3% was reported [15].

Data on the association between MedDiet and sodium intake are scarce [3], particularly among older adults. Therefore, we aimed to quantify the association between sodium excretion and the adherence to the MedDiet among Portuguese older adults.

## 2. Participants and Methods

### 2.1. Study Design and Sampling

A cross-sectional observational study, the Nutrition UP 65 [16], was conducted in Portugal in a sample of 1500 community-dwelling older Portuguese, ≥65 years old, and individuals institutionalized in retirement homes, the latter representing 5% of the sample. To achieve a nationally representative sample of Portuguese older adults, a quota sampling approach was adopted using data from the 2011 Census, regarding sex, age, educational level, and regional area (defined in the Nomenclature of Territorial Units for Statistical purposes—NUTS II). The number of subjects in each stratum of the region, considering the structure of the Portuguese population in terms of sex, age, and education level, was ascertained to obtain a representative sample of the Portuguese population. After randomizing three or more town councils with >250 inhabitants, in each of the seven country areas, potential community dwelling participants who fulfilled the inclusion criteria were invited to participate. They were contacted via home approach, telephone, or via institutions, such as town councils and parish centres. They were recruited until the required numbers of subjects with the relevant characteristics for each stratum of this pre-defined sample were reached. Refusals to participate and their reasons were not registered and, therefore, participation rates were not ascertained. Individuals presenting any condition that precluded the collection of urine, such as dementia or urinary incontinence, were not included.

Data were collected between December 2015 and July 2016. 

### 2.2. Ethics

This research was conducted according to the guidelines established by the Declaration of Helsinki and the study protocol was approved by the Ethics Committee of the department of “Ciências Sociais e Saúde” (Social Sciences and Health) from the “Faculdade de Medicina da Universidade do Porto” (CEDCSS–FMUP 15/2015) and by the Portuguese National Commission of Data Protection (9427/2015). All participants, or two representatives of the participant in cases of cognitive impairment, were asked to read and sign a duplicated “Informed Consent” form.

### 2.3. Data Collection

A structured questionnaire covering socio-demographic data, cognitive performance, physical activity, and nutritional status was applied by eight previously trained registered nutritionists who were also responsible for the anthropometric data collection.

Socio-demographic data included information on sex, age, regional area, education, marital status, and residence type. The regional areas used are defined in NUTS II: Alentejo, Algarve, Azores, Lisbon Metropolitan Area, Center, Madeira and North [17]. Educational level was determined by the number of completed schooling years and the following categories were used: no formal education, 1–3, 4, 5–11, and ≥12 schooling years. Marital status was collected as single, married or in a common-law marriage, divorced, and widowed, and was further categorized as married or single. Residence type was defined as home or institution. The use of diuretics and the presence of chronic diseases, namely hypertension, diabetes, and chronic kidney disease, was self-reported.

The short form of the International Physical Activity Questionnaire (IPAQ) [18], which concerns to activities performed during the past seven days, was used to assess physical activity. Data collected with IPAQ was converted to MET minutes. Median values were calculated for walking, moderate-intensity activities, and vigorous-intensity activities using established formulas. For the total physical activity Metabolic Equivalent of Task (MET), the MET-min/week was defined as the sum of walking + moderate + vigorous MET-min/week scores [19]. MET-minute scores were equivalent to kilocalories for a 60-kg person. Kilocalories were computed from MET-minutes/week scores [18] and individuals were classified as either presenting low physical activity levels, <383 kcal/week (men) and <270/week (women), or as presenting normal physical activity levels (≥383 kcal/week and ≥270/week, respectively, for men and women) [20]. 

Cognitive performance was assessed by the Portuguese version of the Mini Mental State Examination [21]. Cognitive impairment was defined as follows: individuals with no education, ≤15 points; 1 to 11 years of school completed, ≤22 points; and >11 years of school completed, ≤27 points [21].

Each participant’s nutritional status was evaluated by the assessment of anthropometric measurements of body weight and standing height. Body mass index (BMI) was computed using the standard formula (body weight (kg)/standing height^2^ (m)). Overweight and obesity were defined according to WHO cut-off values as BMI (25.00–29.99) and ≥30.00 kg/m^2^, respectively. Undernutrition status was assessed by the Portuguese version of the Mini-Nutritional Assessment^®^-Short Form (MNA^®^-SF) [22,23]. The presence of chronic diseases, namely hypertension, was self-reported.

Anthropometric measurements were collected following standard procedures [24]. Standing height was obtained with a calibrated stadiometer (Seca 213) with 0.1 cm resolution. For participants with visible kyphosis or when it was impossible to measure standing height due to a participant’s paralysis or due to mobility or balance limitations, height was obtained indirectly from non-dominant hand length (in centimetres), measured with a calibrated paquimeter from Fervi Equipment with 0.1 cm resolution [25]. Body weight (in kilograms) was measured with a calibrated portable electronic scale (Seca 803) with 0.1 kg resolution, with the participants wearing light clothes. When it was not possible to weigh a patient, for the same reasons that standing height measurement could not be conducted, body weight was estimated from mid-upper arm and calf circumferences [26]. 

The volume of urine in a 24-h period was collected for each participant. The interviewers gave the participants detailed oral and written instructions (a leaflet was distributed to all participants) on how to proceed for a valid collection and adequate storage of the volume of 24-h urine. Participants were taught to discard the first morning void and to collect all urine over the following 24 h, including the first void on the following morning, and to keep note of the time of the start and end of collection. A 24-h urine 3 L container was also provided. The instructions for the collection of the 24 h urine samples occurred after the interviews. A certified laboratory, Labco Portugal, was responsible for the collection and analyses of urine samples. Analyses included the quantification of urine volume (mL), urinary creatinine (mg/day), and urine sodium (mg/day). Urinary creatinine was measured by the Jaffe method. A urine sample was considered inadequate if the creatinine level was <0.4 g/24-h for women and <0.6 g/24-h for men [27] or if the volume collected was <500 mL [28]. Excessive sodium excretion was defined as ≥2000 mg/day, according to the World Health Organization cut-offs for sodium intake.

The adherence to the MedDiet was evaluated with the Portuguese version of *Prevención com Dieta Mediterránea* (PREDIMED) [29], which consists of 14 questions, each scored with zero or one point. The criteria for assigning one point are established in the questionnaire, and a final score ≥10 indicates a high adherence to the MedDiet [30].

### 2.4. Statistical Analysis

For the present specific analysis, only elderly individuals with valid urine samples were included. Therefore, from the 1500 subjects recruited, 179 participants were excluded because their urine samples were inadequate. A total of 1321 subjects (766 women and 555 men) were considered for data analysis. Socio-demographic and health data were presented as frequencies (n and %) and were compared according to categories of sodium excretion (adequate <2000 vs. excessive ≥2000 mg/day) and according to MedDiet adherence categories (low vs. high adherence) using the Pearson’s chi squared test. 

Descriptive statistics of urinary sodium excretion in mg/day and PREDIMED scores were calculated. The comparison of the medians of sodium excretion according to MedDiet adherence categories (low vs. high adherence) was undertaken using the Mann–Whitney test.

A multivariable binary logistic regression model was conducted to evaluate the association between urinary sodium excretion and adherence to the MedDiet. Odds Ratios (OR) and respective 95% Confidence Intervals (CI) were calculated, adjusted for age, region, type of residence, education level, marital status, use of diuretics, and nutritional status. All data were analysed using Statistical Package for the Social Sciences for Windows, version 24.0. Statistical significance was established at a *p* value < 0.05. 

## 3. Results

Of the 1500 subjects initially recruited, 1321 constituted the final sample; 58% were women, and 26.9% were aged ≥80 years. Regarding the presence of chronic diseases, hypertension, diabetes, and chronic kidney disease were reported, respectively, by 65.0%, 27.3%, and 9.3% of the participants. The use of diuretics was reported by 15.1% of the subjects.

Excessive sodium excretion was observed in 80.0% of men and 91.5% of women, whereas a high adherence to the MedDiet was reported by 42.2% of women and 46.4% of men. 

Table 1 and Table 2 describe the socio-demographic and health characteristics of the participants according to sodium excretion categories (<2000 vs. ≥2000 mg/day) and MedDiet adherence categories (low vs. high adherence). A higher proportion of elderly with excessive sodium excretion was younger (women: 76.7% in those 65–79 years-old vs. 58.7% among women ≥80 years, *p* < 0.001; men: 88.1% in those 65–79 years-old vs. 79.4% in men with ≥80 years, *p* = 0.013), single (women: 81.8% vs. 65.6% among married, *p* < 0.001; men: 89.7% vs. 80.7% in married, *p* = 0.003), not undernourished (only in women: 74.8% vs. 56.2% among those at risk/undernourished, *p* < 0.001), lived at home (only in women: 73.3% vs. 41.7% among those who lived at institutions, *p* < 0.001), and had high physical activity (women: 73.3% vs. 62.5% in those with low physical activity, *p* = 0.010; men: 87.7% vs. 77.8% in those with low physical activity, *p* = 0.012).

Descriptive statistics of urinary sodium excretion (mg/day) and PREDIMED score are presented as supplementary material (Supplementary Table S1). The distribution of medians of sodium excretion according to the categories of Mediterranean Diet adherence are shown in Supplementary Table S2.

High adherence to the MedDiet was more frequent among younger women (45.5% in 65–79 years vs. 33.0% in those ≥80 years, *p* = 0.001), participants with higher education (women: 66.7% in those with >12 schooling years vs. 32.3% among those without schooling, *p* = 0.003; men: 66.7% in those with >12 schooling years vs. 30.2% among those without schooling, *p* = 0.013), single women (49.3% vs. 37.5% in married subjects, *p* = 0.002), and among not-undernourished women (43.7% vs. 33.6% in those at risk/undernourished, *p* = 0.025).

Table 3 presents the odds ratios for the association between the adherence to the MedDiet and adequacy of sodium excretion in the studied sample. After adjusting for potential confounders, excessive sodium excretion was associated with a high adherence to the MedDiet in men (OR = 1.94; 95% CI: 1.03–3.65). No such association was observed in women (OR = 0.91; 95% CI: 0.62–1.34).

## 4. Discussion

In the present study, it was shown that high MedDiet adherence was associated with a higher likelihood of exhibiting higher sodium excretion in males, supporting the hypothesis that salt added during cooking is an important source of sodium, since the foods present in the Mediterranean dietary pattern are not important sources of intrinsic sodium and the consumption of processed foods in the MedDiet is expected to be low. This result contrasts with the findings of the SUN study conducted in a sample of Spanish adults, in which a decrease in sodium intake with increasing quintiles of adherence to the MedDiet was observed. However, the authors highlighted that only individuals with a high level of education were included in the study, which is associated with a better dietary pattern. In addition, in the latter work, sodium intake was assessed by food frequency questionnaire, while in the present study, 24-h urinary sodium excretion was used, which is considered the gold standard to assess the intake of this nutrient [31].

Throughout most of the world, dietary sodium is well above the recommendations, and cardiovascular disease is the leading cause of death in most developed countries. It is, therefore, essential to evaluate the dietary sources of this nutrient in order to design strategies to reduce its intake. In more developed countries such as those in Europe and North America, sodium added by the food industry to processed foods is the main source of sodium while, in less developed regions or in populations with rooted cooking habits where gastronomy plays a relevant role, including South Africa, Mozambique, and China, the main sources of sodium include the salt added to food in cooking [32,33,34,35,36,37,38]. Thus, efforts to reduce sodium intake may benefit from campaigns to raise awareness among the population in terms of cooking with less added salt and not adding salt at the table. In fact, Idelson et al. supported this strategy since they found an interrelation between salt knowledge and behaviour, and both were significantly and directly related to the degree of adherence to a MedDiet [39]. In line with the latter work, the results of the present study showed a positive association between education and adherence to the MedDiet in both sexes, reflecting the effect of education on food choice.

In addition to discretionary sodium, the MedDiet may also include processed foods with non-negligible sodium contents, such as cheese, olives, and bread [3]. Therefore, the role of the food industry in minimizing the salt added during processing together with legislation regulating the salt limit in commercial foods is essential. It is estimated that decreasing dietary salt intake from the current global levels of 9–12 g per day—to the recommended level of up to 5 g per day—would have an important impact in terms of reducing blood pressure and cardiovascular diseases [40].

Compared to women, men more frequently add salt to the table or more often choose processed foods with sizable salt contents. In fact, it was reported that women are more likely than men to report limiting their consumption of salt and processed foods [41,42], which may contribute to explaining the observed association between sodium excretion and MedDiet adherence only in men.

At first sight, the inverse association between MedDiet adherence and sodium intake adequacy seems paradoxical since the MedDiet has been described as being inversely associated with hypertension, which is, in turn, related to excessive sodium intake [6,8,9,40,43,44]. However, a recent study conducted in a Mediterranean cohort to assess the relation between the adherence to the MedDiet and the occurrence of hypertension showed that this inverse association loses statistical significance after adjustment for sodium and potassium intake [45], suggesting that salt may be a key variable that mediates the effects of the Mediterranean Diet with regard to disease impact.

The study of the impact of the MedDiet on the health of the populations was initiated by Ancel Keys in 1952 to understand the reduced rates of cardiovascular disease in this region. According to Keys, the focus of the MedDiet definition was based on low contents of saturated fats and high contents of vegetable oils, observed in Greece and Southern Italy during the 1960s. Over the last decades, the definition originally introduced by Keys has evolved and diverse definitions were subsequently proposed, based on nutritional contents and foods consumed, or using a priori or a posteriori scoring systems [46]. In addition, the instruments used to assess the adherence to MedDiet are diverse. The original index of adherence to the MedDiet was published in 1995 in Greece and, since then, several adapted versions were settled for different population groups. The PRediMed instrument, developed in Spain with the objective of testing the efficacy of the MedDiet in the primary prevention of cardiovascular disease, has been widely used because it is easily applicable in clinical contexts. As with most indexes, it does not contemplate the use of salt in cooking and at the table, or the intake of salty foods [29], which is a limitation when assessing cardiovascular risk. 

A limitation of this study was the single period of 24-h urine collection, which may not represent an individual’s normal behaviour. Nonetheless, this may also be considered a strength of the study since the 24-h urine collection is considered to be the gold standard in the assessment of sodium excretion. The available resources and the timeline of the Nutrition UP 65 project did not allow the inclusion of the participants’ dietary intake assessment, thus preventing the estimation of sodium sources, which would be important in defining strategies to reduce sodium intake in older populations. 

## Figures and Tables

**Table 1:** Socio-demographic and health characteristics of female participants, by sodium excretion and Mediterranean Diet adherence.

	Sodium Excretion			MedDiet Adherence		
	Adequate (<2000 mg/day)	Excessive (≥2000 mg/day)	*p*	Low (<10 Points)	High (≥10 Points)	*p*
*N*	220	546		446	320	
Age (years)						
65–79, *n* (%)	125 (23.2%)	411 (76.7%)	<0.001	292 (54.5%)	244 (45.5%)	0.001
≥80, *n* (%)	95 (41.3%)	135 (58.7%)		154 (67.0%)	76 (33.0%)	
Education (schooling years)						
0, *n* (%)	49 (36.8%)	84 (63.2%)	0.095	90 (67.7%)	43 (32.3%)	0.003
<4, *n* (%)	55 (31.8%)	118 (68.2%)		109 (63.0%)	64 (37.0%)	
4, *n* (%)	90 (25.5%)	263 (74.5%)		194 (55.0%)	159 (45.0%)	
5–12, *n* (%)	19 (24.7%)	58 (75.3%)		43 (55.8%)	34 (44.2%)	
>12, *n* (%)	7 (23.3%)	23 (76.7%)		10 (33.3%)	20 (66.7%)	
Residence						
Home, *n* (%)	192 (26.7%)	526 (73.3%)	<0.001	417 (58.1%)	301 (41.9%)	0.750
Institution, *n* (%)	28 (58.3%)	20 (41.7%)		29 (60.4%)	19 (39.6%)	
Marital Status						
Married, *n* (%)	169 (34.4%)	322 (65.6%)	<0.001	307 (62.5%)	184 (37.5%)	0.002
Single, *n* (%)	50 (18.2%)	224 (81.8%)		139 (50.7%)	135 (49.3%)	
Physical Activity						
Low (<270 kcal/week), *n* (%)	54 (37.5%)	90 (62.5%)	0.010	89 (61.8%)	55 (38.2%)	0.334
High (≥270 kcal/week), *n* (%)	166 (26.7%)	456 (73.3%)		357 (57.4%)	265 (42.6%)	
Nutritional Status (MNA)						
Not undernourished, *n* (%)	156 (25.2%)	464 (74.8%)	<0.001	349 (56.3%)	271 (43.7%)	0.025
At Risk/Undernourished, *n* (%)	64 (43.8%)	82 (56.2%)		97 (66.4%)	49 (33.6%)	
BMI						
Normal (<25 kg/m^2^), *n* (%)	27 (26.2%)	76 (73.8%)	0.505	59 (57.3%)	44 (42.7%)	0.326
Overweight (25–29.9 kg/m^2^), *n* (%)	98 (27.7%)	256 (72.3%)		216 (61.0%)	138 (39.0%)	
Obesity (≥30 kg/m^2^), *n* (%)	92 (31.2%)	203 (68.8%)		163 (55.3%)	132 (44.7%)	

MedDiet, Mediterranean Diet; MNA, Mini-Nutritional Assessment^®^-Short Form; BMI, Body Mass Index.

**Table 2:** Socio-demographic and health characteristics of male participants, by sodium excretion and Mediterranean Diet adherence.

	Sodium Excretion			MedDiet Adherence		
	Adequate (<2000 mg/day)	Excessive (≥2000 mg/day)	*p*	Low (<10 Points)	High (≥10 Points)	*p*
*N*	77	478		306	249	
Age (years)						
65–79, *n* (%)	51 (11.9%)	378 (88.1%)	0.013	237 (55.2%)	192 (44.8%)	0.924
≥80, n (%)	26 (20.6%)	100 (79.4%)		69 (54.8%)	57 (45.2%)	
Education (schooling years)						
0, *n* (%)	8 (15.1%)	45 (84.9%)	0.843	37 (69.8%)	16 (30.2%)	0.013
<4, *n* (%)	12 (16.2%)	62 (83.8%)		42 (56.8%)	32 (43.2%)	
4, *n* (%)	43 (14.2%)	259 (85.8%)		171 (56.6%)	131 (43.4%)	
5–12, *n* (%)	10 (10.4%)	86 (89.6%)		46 (47.9%)	50 (52.1%)	
>12, *n* (%)	4 (13.3%)	26 (86.7%)		10 (33.3%)	20 (66.7%)	
Residence						
Home, *n* (%)	72 (13.4%)	466 (86.6%)	0.060	299 (55.6%)	239 (44.4%)	0.240
Institution, *n* (%)	5 (29.4%)	12 (70.6%)		7 (41.2%)	10 (58.8%)	
Marital Status						
Married, *n* (%)	43 (19.3%)	180 (80.7%)	0.003	134 (60.1%)	89 (39.9%)	0.059
Single, *n* (%)	34 (10.3%)	297 (89.7%)		172 (52.0%)	159 (48.0%)	
Physical Activity						
Low (<383 kcal/week), *n* (%)	20 (22.2%)	70 (77.8%)	0.012	52 (57.8%)	38 (42.2%)	0.582
High (≥383 kcal/week), *n* (%)	57 (12.3%)	408 (87.7%)		254 (54.6%)	211 (45.4%)	
Nutritional Status (MNA)						
Not undernourished, *n* (%)	65 (13.4%)	421 (86.6%)	0.366	265 (54.5%)	221 (45.5%)	0.444
At Risk/Undernourished, *n* (%)	12 (17.4%)	57 (82.6%)		41 (59.4%)	28 (40.6%)	
BMI						
Normal (<25 kg/m^2^), *n* (%)	9 (10.2%)	79 (89.8%)	0.488	55 (62.5%)	33 (37.5%)	0.332
Overweight (25–29.9 kg/m^2^), *n* (%)	35 (15.4%)	193 (84.6%)		122 (53.5%)	106 (46.5%)	
Obesity (≥30 kg/m^2^), *n* (%)	33 (14.7%)	191 (85.3%)		122 (54.5%)	102 (45.5%)	

MedDiet, Mediterranean Diet; MNA, Mini-Nutritional Assessment^®^-Short Form; BMI, Body Mass Index.

**Table 3:** Odds ratios for the association between adherence to the Mediterranean Diet and adequacy of sodium excretion in Portuguese elderly.

	Females		Males	
	Adequate Sodium Excretion	Excessive Sodium Excretion	Adequate Sodium Excretion	Excessive Sodium Excretion
	Reference	OR (95% CI) *	Reference	OR (95% CI) *
MedDiet (High Adherence vs. low adherence)	1	0.91 (0.62–1.34)	1	1.94 (1.03–3.65)

OR, odds ratio; 95% CI, 95% confidence interval. * Models adjusted for age, region, type of residence, education level, marital status, use of diuretics, and nutritional status.

## Data Availability

The data presented in this study are available on request from the corresponding author.

## Supplementary Materials

The following are available online at https://www.mdpi.com/article/10.3390/nu14010061/s1, Table S1: Descriptive statistics of urinary sodium excretion (mg/day) and PREDIMED score, Table S2: Medians of sodium excretion according to categories of Mediterranean Diet adherence.
