# Supplementary material for: Urinary Sodium Excretion and Adherence to the Mediterranean Diet in Older Adults
Source: Nutrients. 2021 Dec 24;14(1):61. doi: 10.3390/nu14010061 (PMC8747036; doi:10.3390/nu14010061)
Supplement: Supplementary file 1 [file nutrients-14-00061-s001.zip › nutrients-1486315-SI.pdf]

Supplementary Table S1. Descriptive statistics of urinary sodium excretion (mg/day) and PREDIMED score

Sodium excretion (mg/day)		
	Women	Men
Mean	2937	3807
Percentile 25	2092	2736
Percentile 50	2782	3678
Percentile 75	3609	4667
**Adherence to the Mediterranean Diet (PREDIMED score)**		
	Women	Men
Mean	9.06	9.12
Percentile 25	8	8
Percentile 50	9	9
Percentile 75	10	11

Supplementary Table S2. Medians of sodium excretion according to categories of Mediterranean Diet adherence.

**Sodium excretion (median mg/day)**			
	Low Adherence to MedDiet	High Adherence to MedDiet	P value
Women	2944	2633	0.004
Men	3588	3749	0.182
